# Data Resource Profile: Yorkshire Specialist Register of Cancer in Children and Young People (Yorkshire Register)

**DOI:** 10.1093/ije/dyac195

**Published:** 2022-10-13

**Authors:** Kirsten J Cromie, Paul Crump, Nicola F Hughes, Sarah Milner, Diana Greenfield, Anna Jenkins, Richard McNally, Dan Stark, Charles A Stiller, Adam W Glaser, Richard G Feltbower

**Affiliations:** Leeds Institute for Data Analytics, School of Medicine, Clinical and Population Sciences Department, University of Leeds, Leeds, UK; Leeds Institute for Data Analytics, School of Medicine, Clinical and Population Sciences Department, University of Leeds, Leeds, UK; Leeds Institute of Medical Research, School of Medicine, University of Leeds, Leeds, UK; Leeds Institute of Medical Research, School of Medicine, University of Leeds, Leeds, UK; Sheffield Children's NHS Foundation Trust, Haematology and Oncology Department, Sheffield, UK; Sheffield Children's NHS Foundation Trust, Haematology and Oncology Department, Sheffield, UK; Population Health Sciences Institute, Faculty of Medical Sciences, Newcastle University, Newcastle upon, UK; Leeds Institute of Medical Research, School of Medicine, University of Leeds, Leeds, UK; National Disease Registration Service, NHS Digital, England; Leeds Institute for Data Analytics, School of Medicine, Clinical and Population Sciences Department, University of Leeds, Leeds, UK; Leeds Institute of Medical Research, School of Medicine, University of Leeds, Leeds, UK; Leeds Institute for Data Analytics, School of Medicine, Clinical and Population Sciences Department, University of Leeds, Leeds, UK

Key Features

- The Yorkshire Register is an established population-based research database of tumours diagnosed in the childhood, adolescent and young-adult age ranges.^1^ Set up in 1984 to describe medium- and long-term outcomes for cancer survivors, it remains one of only three specialist databases in the UK covering the paediatric age range and the only one of its kind covering individuals diagnosed aged 25–29 years.
- The Yorkshire Register is held on an encrypted firewall-protected secure platform at the University of Leeds.^2^ Individuals diagnosed with a malignant or benign central nervous system tumour aged 0–29 years whilst living in the area contiguous with the former Yorkshire and the Humber Strategic Health Authority^3^ are eligible for inclusion. Information has been collected for children diagnosed since 1974. Data accrual for 15- to 29-year-olds began in 1990.
- The Yorkshire Register collects data prospectively and currently contains information on 11 702 primary and secondary tumours. Data have been linked to various administrative health-related (Hospital Episode Statistics,^4^ National Cancer Registration and Analysis Service^5^) and non-health-related (National Pupil Database^6^) data sets.
- Personal and demographic information along with diagnostic and clinical data on treatment, death information and date last seen in follow-up clinics are available for all individuals.
- Other research teams (subject to review, with the appropriate ethical and information governance approvals^7^) can use the data held in the Yorkshire Register for the purposes of further research.

## Data resource basics

Cancer is a rare disease in children and young people^8^^,^^9^ and is commonly defined such that it includes any malignant tumour or benign central nervous system (CNS) tumour. An average of 1645 cases of childhood cancer (0–14 years) are diagnosed each year in the UK and ∼2110 per year for 15- to 24-year-olds.^8^ Registrations of newly diagnosed cases of cancer in individuals aged 0–29 years account for just 1–2% of all cancer registrations in England.^10^ Despite this, cancer places a considerable burden not only upon the individuals themselves but also on their families and the healthcare system.^10^^,^^11^ Much remains unknown about the aetiology and long-term outcomes of cancer in this young age group.^10^

The Yorkshire Specialist Register of Cancer in Children and Young People (Yorkshire Register)^1^ was established in 1984 to address this deficit, providing a basis for world-leading epidemiological and outcomes research examining the patterns and causes of cancer in children and young people and undertaking applied health services research generating novel insights into cancer outcomes. The Yorkshire Register specifically aims to collect comprehensive diagnostic, treatment and outcomes information that adds value and is not readily available from other routine National Health Service (NHS) sources, including the National Cancer Registration Analysis Service (NCRAS).^5^ Data from individuals held in the Yorkshire Register are linked to a suite of administrative health and non-health-related data sources including secondary care [Hospital Episode Statistics^3^ (HES)], death registration records and the National Pupil Database (NPD)^6^ using NHS number, date of birth, sex and patient postcode at diagnosis.

The Yorkshire Register is held on an encrypted firewall-protected secure platform within Leeds Institute for Data Analytics at the University of Leeds.^2^ Subject to ongoing research funding, the data will be held indefinitely enabling the accrual of an ever-increasing data set relating to cancer in young people and allowing more powerful statistical comparisons to be performed.

**Figure 1:**
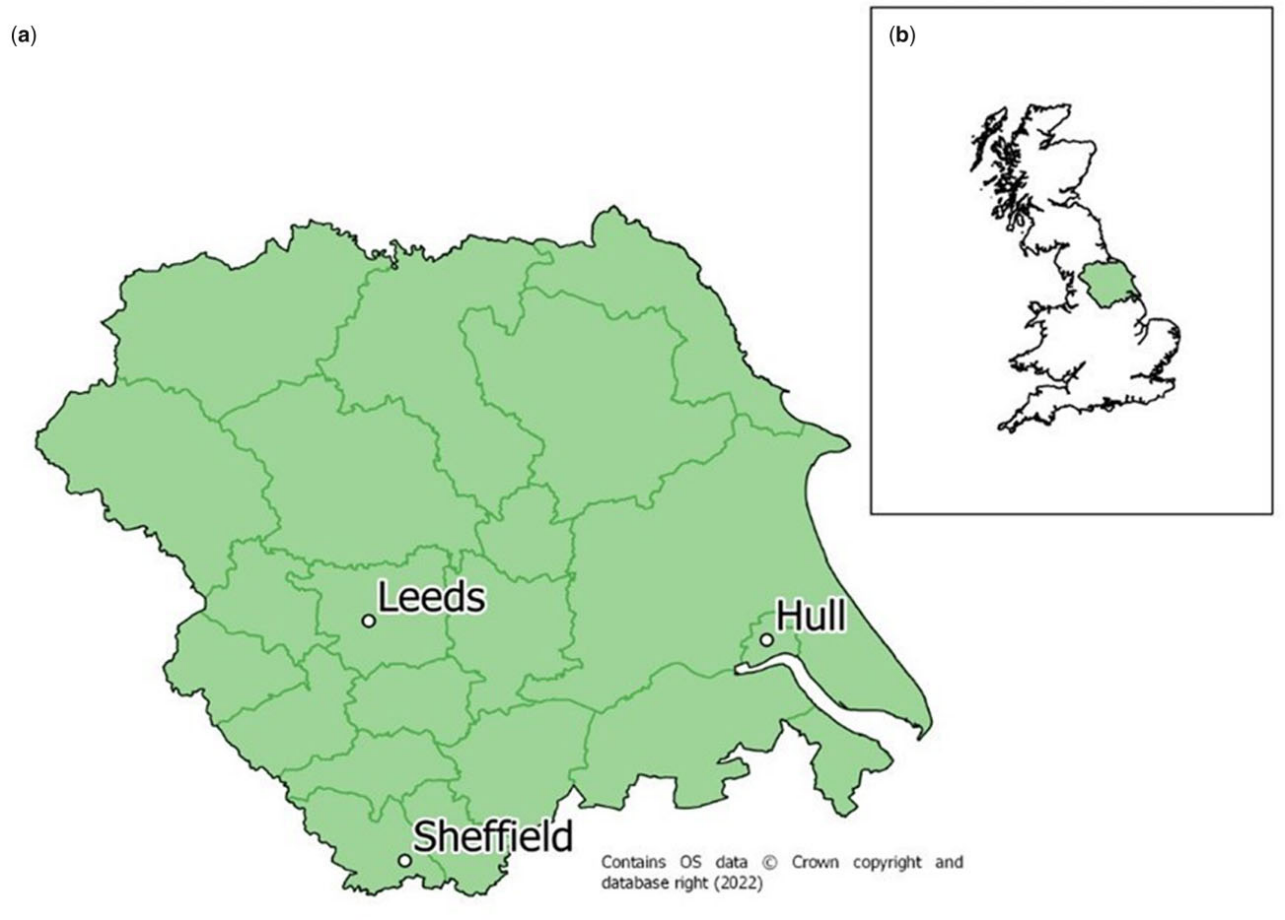
Yorkshire Specialist Register of Children and Young People (Yorkshire Register) population coverage. (a) Map of Yorkshire & the Humber; (b) map of the UK with Yorkshire & the Humber highlighted.

The Yorkshire Register is a regional population-based research database of cancer diagnoses. The geographical area covered by the Yorkshire Register is contiguous with the former Yorkshire and the Humber Strategic Health Authority (Yorkshire, UK) (Figure 1).^2^ The region covers a population of 5.5 million, 2 million of whom are aged <30 years.^12^ Individuals aged <30 years diagnosed with a primary malignant (or benign CNS) tumour whilst resident in the area are eligible for inclusion on the Yorkshire Register, even if they are diagnosed outside of the Yorkshire area.

The Yorkshire region comprises a diverse mix of urban and rural communities, with a significant ethnic minority population that is predominantly of south Asian origin, comprising 6% of the Yorkshire & the Humber population in the 2011 Census.^13^ An estimated 60% of the south Asian population in Yorkshire also originates from Mirpur in rural Pakistan.^14^ This makes Yorkshire one of the few regions in the UK that allows detailed analysis of a relatively homogeneous, second- and third-generation south Asian population.

As of 15 February 2022, the Yorkshire Register held data on 11 702 tumours diagnosed in 11 482 individuals, representing ∼0.6% of the population of under-30-year-olds in England.^12^ Eight thousand four hundred and ninety-five (74%) of those individuals were alive at the time of data extraction (Table 1).

Key demographic information on all individuals held in the Yorkshire Register is presented in Table 1. At the time of extraction, 83.3% of individuals were White, just over 10% were Asian and just under 1% were Black. Yorkshire Register patient ethnicity is based primarily on data from linked HES Admitted Patient Care (APC),^2^ which records ethnic groups based on 1991/2001 Census categories depending on date of hospital admission (Table 1).^16^ Where this information was missing historically, results from Onomap naming algorithms were used.^17^ Yorkshire Register patient ethnicity assignment has been described in detail previously.^17^ Combining ethnicity data from multiple sources ensures complete ethnicity data are available for all individuals in the Register where possible.

**Table 1:** Demographic characteristics^a^ of the Yorkshire Specialist Register of Cancer in Children and Young People cohort, February 2022

	0–14 years	15–29 years	0–29 years
	1974–present (%)	1990–present (%)	1974/1990–present
Total number of individuals	5317	6165	11 482
Total alive	3621 (68.1)	4678 (79.0)	8495 (74.0)
Sex			
Female	2353 (44.2)	2490 (40.4)	4843 (42.2)
Male	2964 (55.8)	3675 (59.6)	6639 (57.8)
Age at diagnosis (years)			
0–4	2466 (46.3)	–	2466 (21.5)
5–9	1412 (26.6)	–	1412 (12.3)
10–14	1439 (27.1)	–	1439 (12.5)
15–19	–	1433 (23.2)	1433 (12.5)
20–24	–	2041 (33.1)	2041 (17.8)
25–29	–	2691 (43.7)	2691 (23.4)
Ethnicity (1991 Census categories)^b^			
White	4438 (83.5)	5086 (82.4)	9524 (82.9)
Black	46 (0.9)	53 (0.9)	99 (0.9)
Indian	32 (0.6)	40 (0.7)	72 (0.6)
Pakistani	340 (6.4)	332 (5.4)	672 (5.9)
Bangladeshi	13 (0.2)	15 (0.2)	28 (0.2)
Chinese	3 (0.1)	18 (0.3)	21 (0.2)
Other	184 (3.5)	206 (3.3)	390 (3.4)
Not stated^c^	262 (4.9)	420 (6.8)	682 (5.9)
Townsend Index of Deprivation score^15^			
1 (least deprived)	866 (16.3)	939 (15.9)	1852 (16.1)
2	974 (18.3)	1006 (17.0)	2028 (17.7)
3	973 (18.3)	1182 (20.0)	2194 (19.1)
4	1009 (19.0)	1169 (19.7)	2223 (19.4)
5 (most deprived)	1439 (27.1)	1564 (26.4)	3070 (26.7)
Unknown	57 (1.1)	62 (1.1)	121 (1.05)

a Patient demographics are based on diagnostic information from first primary registerable tumour.

b Yorkshire Register ethnicity is based primarily on data from linked Hospital Episode Statistics Admitted Patient Care,^2^ which records ethnic groups based on 1991/2001 Census categories based on date of diagnosis (Table 1).^16^ Where this information was missing historically, results from Onomap naming algorithms were used.^17^

c Ethnicity information was not available from electronic health records, linked Hospital Episode Statistics data or *Onomap* naming algorithms.^17^

Townsend deprivation index is used as a measure of area-based material deprivation for each individual.^15^ The index is based on official statistics on rates of unemployment, non-home ownership, household over-crowding and non-car ownership (Supplementary Box S1, available as Supplementary data at *IJE* online); 27% of children (0–14 years) and 26% of 15- to 29-year-olds are in the most deprived fifth (V). When compared with 20% for England as a whole, this is indicative of a slightly more deprived population in the Yorkshire childhood and young-adult cancer population.

## Data collected

### Core data collection

A copy of the Data Collection Form can be found on the Yorkshire Register website (https://ysrccyp.org.uk/wp-content/uploads/sites/103/2021/07/Protocol-April-2021-Data-Collection-Form.pdf).

**Figure 2:**
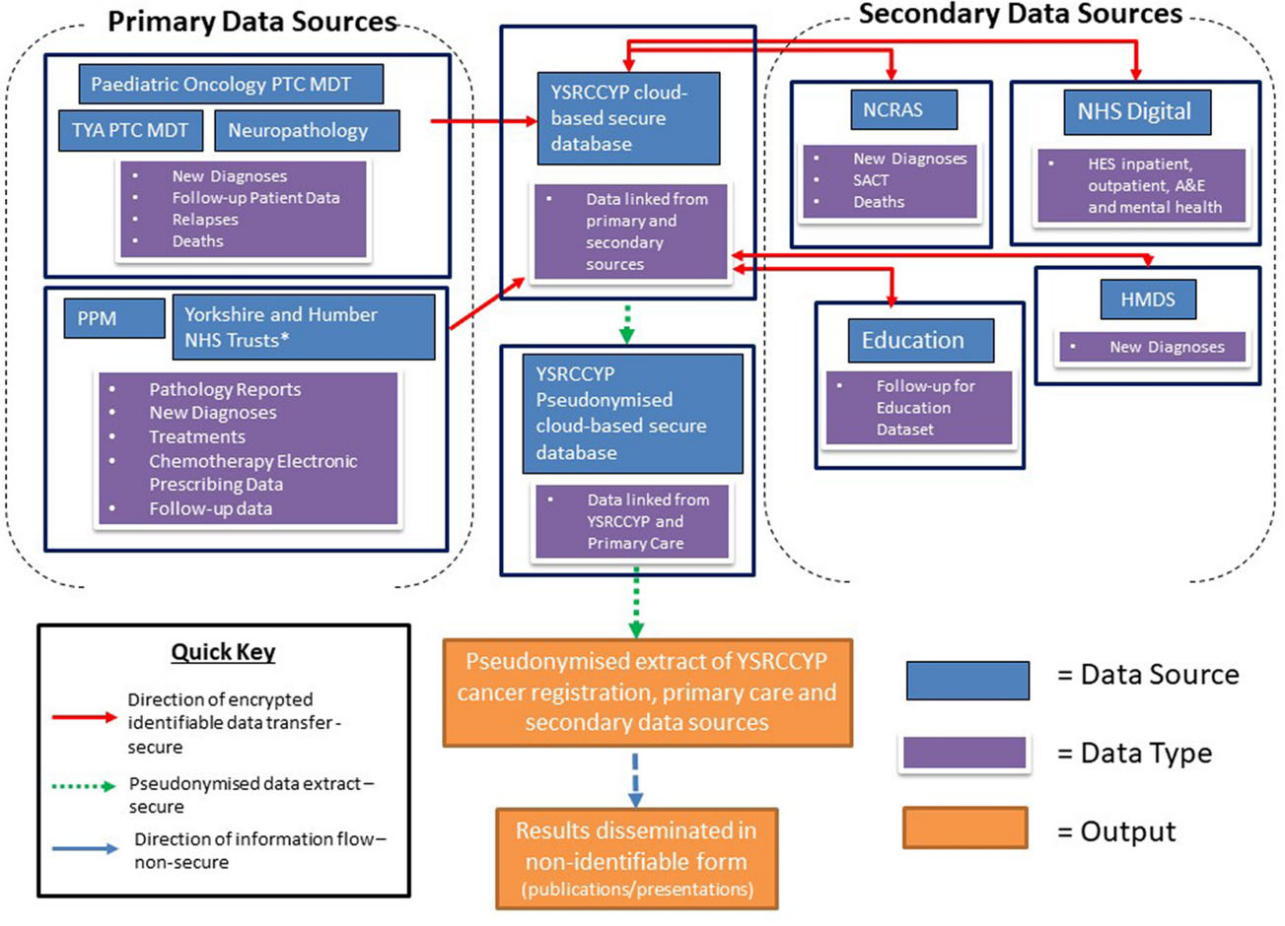
Yorkshire Specialist Register of Cancer in Children and Young People (Yorkshire Register) Data Collection Flow overview (February 2022) HES, Hospital Episode Statistics; HMDS, Haematological Malignancy Diagnostic Service; LTH, Leeds Teaching Hospitals; MDT, Multidisciplinary Team; NCRAS, National Cancer Registration Service; PHE, Public Health England; PTC, Principal Treatment Centre; PPM/PPM+, Patient Pathway Manager (LTH electronic patient information database); SACT, Systemic anti-cancer therapies data set; TYA, Teenage and Young Adult. ^a^A full list of Yorkshire and Humber NHS Trusts can be found in Supplementary Box S2 (available as Supplementary data at *IJE* online).

The primary source of notifications for paediatric tumours are the two Principal Treatment Centres in Yorkshire [Leeds Teaching Hospitals Trust (LTHT) and Sheffield Children’s Hospital NHS Foundation Trust (SCH)]. For 16- to 24-year-olds, the primary source is the teenage and young-adult multidisciplinary teams at LTHT and Sheffield Teaching Hospitals NHS Foundation Trust. For all other ages (25–29 years), the primary source of notifications is NCRAS. An electronic feed has been established from local hospital patient management and pathology systems [including Patient Pathway Manager (PPM)^18^ in Leeds, with a view to expand across all NHS Trusts across Yorkshire], as well as NCRAS^5^ to improve the efficiency of the Yorkshire Register data collection. The Neuropathology department at LTHT provides an additional secondary source of notifications for all 0- to 29-year-olds referred for diagnosis and/or treatment and the Haematological Malignancy Diagnostic Service for all haematological tumours (Figure 2). Where essential data on cancer diagnosis and treatment are missing or incomplete from the electronic data sources, information is manually abstracted from local hospital notes and patient management systems. Yorkshire Register data are cross-checked annually and validated against other data sets held by the NCRAS^5^ for the purposes of quality assurance.

All addresses and postcodes at diagnosis are verified using the Office for National Statistics (ONS) National Statistics Postcode Lookup^19^ to ensure geographical eligibility. Each postcode is mapped to a small area census code and assigned to a census enumeration district or Output Area, and then aggregated up into lower super output areas, county districts, counties or Clinical Commissioning Groups within the Yorkshire region, dependent on the geographical level of analysis. This permits the characterization of geographical areas by social class, ethnic group and other variables such as population migration at different scales using census data.

### Additional registration details

All diagnoses on the Yorkshire Register are coded according to ICD-O versions 2 and 3 (based on ICD-10/ICD-11) using morphology and site, and grouped according to the International Classification of Childhood Cancer (third edition) (ICCC-3)^20^ (Table 2). Copies of diagnostic pathology reports, cytogenetic and molecular genetic diagnostics are retained to provide comprehensive information on diagnosis and facilitate future research should diagnostic classifications change.

**Table 2:** Clinical and diagnostic characteristics^a^ of Yorkshire Specialist Register of Cancer in Children and Young People cohort, February 2022

	0–14 years	15–29 years	0–29 years
	1974–present (%)	1990–present (%)	1974/1990–present
ICCC-3 Diagnostic group^22^			
I. Leukaemias, myeloproliferative diseases and myelodysplastic diseases	1612 (30.3)	587 (9.5)	**2199 (19.2)**
II. Lymphomas and reticuloendothelial neoplasms	607 (11.4)	1424 (23.1)	**2031 (17.7)**
III. CNS and miscellaneous intracranial and intraspinal neoplasms	1298 (24.4)	720 (11.7)	**2018 (17.6)**
IV. Neuroblastoma and other peripheral nervous cell tumours	369 (6.9)	20 (0.3)	**389 (3.4)**
V. Retinoblastoma	158 (3.0)	–	**158 (1.4)**
VI. Renal tumours	296 (5.6)	67 (1.1)	**363 (3.2)**
VII. Hepatic tumours	66 (1.2)	42 (0.7)	**108 (0.9)**
VIII. Malignant bone tumours	252 (4.7)	235 (3.8)	**487 (4.2)**
IX. Soft tissue and other extraosseous sarcomas	350 (6.6)	315 (5.1)	**665 (5.8)**
X. Germ cell tumours, trophoblastic tumours and neoplasms of gonads	183 (3.4)	1419 (23.0)	**1602 (14.0)**
XI. Other malignant epithelial neoplasms and malignant melanomas	115 (2.2)	1312 (21.3)	**1427 (12.4)**
XII. Other and unspecified malignant neoplasms	11 (0.2)	24 (0.4)	**35 (0.3)**
Total number of tumours			
1	5197 (96.6)	6081 (96.2)	11 278 (96.4)
2^b^	171 (3.2)	207 (3.3)	378 (3.2)
3^b^	12 (0.2)	30 (0.5)	42 (0.4)
4^b^	–	4 (0.1)	4 (<0.1)
At least one relapse			
Yes	856 (16.1)	496 (8.0)	1352 (11.8)

a Based on first primary registerable tumour.

b SMNs are defined as a malignant neoplasm of any site with a different morphology from that of the primary tumour regardless of time since diagnosis according to the recommended coding of multiple primary cancers.^21^^,^^22^

ICCC-3, International Classification of Childhood Cancer (third edition)^20^; CNS, central nervous system.

Notifications of relapse (including date of relapse) and secondary malignant neoplasms (SMNs) are obtained via flagging and linkage with NCRAS.^5^ If a patient is diagnosed with a SMN while under the age of 25 years and resident in Yorkshire, then this information is normally also acquired directly from the principle treatment centres (LTHT and SCH) in Yorkshire as part of the core data collection (Table 2). Data received from NCRAS includes date of subsequent tumour diagnosis and diagnostic group, coded to ICD-O-2 or ICD-O-3 (depending on the date of diagnosis). SMNs are defined according to the recommended coding of multiple primary cancers.^21^^,^^22^

### Additional data linkages (enhanced treatment information and death notifications)

Enhanced treatment information on chemotherapy and radiotherapy is obtained through linkage with NCRAS,^5^ including the national Systemic Anti-Cancer Therapy (SACT)^23^ and Radiotherapy data sets,^24^ as well as hospital electronic prescribing systems such as ChemoCare.^25^ ChemoCare extracts are updated annually and SACT every 2 years for use as part of continual data validation exercises for the Register and to facilitate a programme of research comparing the chemotherapy doses and intensities given to individuals and subsequent effect on outcomes. Figure 2 presents the Yorkshire-wide data flow process describing the linkage of Yorkshire Register data with other registry and healthcare databases.

Follow-up information is derived from electronic feeds from PPM^18^ and NCRAS^5^ (Figure 2). The Yorkshire Register is linked to ONS Death Registration Data,^26^ considered the gold standard for mortality data in the UK.^27^ Information is provided for deaths occurring in England, whether the individuals are dead, embarked or untraceable; death certificates are also sent to us listing cause of death and place of death (Table 3). Currently we estimate that <0.1% of all individuals on the Yorkshire Register have been lost to follow-up based on the aforementioned cross-checking with NCRAS and the Personal Demographics Service (NHS Digital).^28^

**Table 3:** Standard linkages with the Yorkshire Specialist Register of Cancer in Children and Young People research database

Linkage data set	Coverage	Key information
ONS Death Registration Data	1974/1990–2019	Date, place, and cause of death (ICD)
**NHS Digital**		
HES Admitted Patient Care (APC)	1996–2021	Patient information including ethnic group^a^
		Diagnoses (ICD) and procedures (OPCS)
HES Outpatient Care	2003–2021	Diagnoses (ICD) data
Accident & Emergency (A&E)	2007–2021	Diagnoses (A&E codes) data
Mental Health Services Dataset (MHSDS)^29^	2006–2021	Diagnoses (ICD) and care received (OPCS)
**Public Health England (PHE)**		
National Cancer Registration and Analysis Service (NCRAS)		Diagnoses (ICD) and tumour site
Systemic Anti-Cancer Treatment (SACT)	2012–2019	Procedures and outcomes (ICD & OPCS)
National Radiotherapy Dataset	2015–2019	Procedures (ICD & OPCS)
**Department of Education (DfE)**		
National Pupil Database (NPD)	2001–2020/21	Educational outcomes (KS2–KS5), SEN data, pupil absences
**Small area-level data (LSOA)**		
Townsend Deprivation Index^15^	1971, 1981, 1991, 2001, 2011	LSOA-level deprivation data based on patient postcode at diagnosis
Leeds Teaching Hospitals Trust (PPM)^18^	2002–2021	Specific risk markers for cardiovascular disease plus factors associated with metabolic syndrome and Type II diabetes e.g. HbA1c, lipid profile, blood pressure and anthropometric measurements
		Holistic needs assessment

ONS, Office for National Statistics; ICD, International Classification of Diseases; OPCS, Office of Population Censuses and Surveys Classification of Interventions and Procedures; KS, key stage; PPM, Patient Pathway Manager; HbA1c, haemoglobin A1C; HES, Hospital Episode Statistics; NHS, National Health Service.

a According to Office for National Statistics 2001 Census categories.^16^

## Data resource use

Linking information on outcomes from secondary care (obtained from HES data including APC, outpatient, accident and emergency, and mental health admissions) has facilitated a range of published studies into aetiology, patterns of care and treatment vs outcomes.^22^^,^^30–37^ Linked HES APC data have been used to investigate the long-term sequelae of cardiovascular disease,^22^^,^^32^ respiratory morbidity^30^ and SMNs.^20^

The Yorkshire Register has been instrumental in supporting a body of research that identified poor treatment outcomes for cancer in teenagers and young adults aged 13–24 years.^38^ Findings had major implications in substantiating new NHS policy in 2005, which led to the introduction of specialized teenager and young-adult cancer services. From 2016 onwards, this has led to gradual improvement in the health outcomes for teenagers and young adults.^37^

There are plans to continue the internationally recognized programme of research on childhood and young-adult cancer outcomes using the Yorkshire Register database. Current and future projects include investigations into mental health outcomes, fertility problems and cardiometabolic diseases in the long-term survivor population. We aim to identify how these outcomes vary by demographic factors as well as type of malignancy and treatment received. Through novel data linkage to the National Pupil Database^6^ (Table 2) we also plan to investigate how the educational trajectory of individuals is affected by a cancer diagnosis in the childhood and young-adult years.

A bibliography of all peer-reviewed published studies using Yorkshire Register data can be found in the Appendix.

## Strengths and weaknesses

With detailed demographic, clinical and follow-up data on >11 000 individuals stretching back almost 50 years and linkages to multiple NHS and other routine data sets, the Yorkshire Register research database provides an invaluable and unique population-based data resource for researchers, clinicians and commissioners to further understand the causes and outcomes of cancer in young people.

The accuracy, completeness and comprehensiveness of the clinical and socio-demographic data held on children, teenagers and young adults with cancer in Yorkshire is very strong. The Yorkshire Register is the only specialist database of its kind in England that covers all individuals diagnosed with cancer under the age of 30 years. The demographic and ethnic profile of Yorkshire, in conjunction with validated postcode at diagnosis and the ascertainment of complete and accurate ethnicity data from multiple sources, enables us to explore crucial differences in incidence and prognosis for specific ethnic minority groups or individuals from socio-economically deprived areas^36–38^ where national estimates are unavailable.^41^ Whilst the data collected are limited to the Yorkshire region, intelligence generated is of benefit to national and international health service and research partners with whom we increasingly collaborate to support improvements in healthcare and cancer outcomes.

Innovative data linkages with routinely collected administrative data facilitate the Yorkshire Register’s world-leading programme of research on childhood and young-adult cancer outcomes, extending into ground-breaking areas of social and psychosocial morbidity. Internationally, the data linkages employed by the Yorkshire Register have been recognized as exemplary methods of evaluating patient outcomes using routine health data sets.^40^

There are some limitations: for example, given the rarity of certain diagnostic groups, limited analyses are possible using data held on this regional resource due to small numbers. This can hinder the extent to which detailed subgroup analyses can be undertaken. Data are not available for older teenagers and young adults aged 15–29 years diagnosed before 1990 nor linked HES APC data prior to 1996.

## Data resource access

Upon receipt of a Data Access Request Form, requests are considered by the Registry Director and Medical Director, the Chair of the Yorkshire Register Scientific Advisory Group,^41^ the Registry Data Manager and an independent representative from the LIDA Data Analytics Team, together forming the ‘Registry Data Release Panel’. Research proposals will be circulated to all Scientific Advisory Group members (including external expertise) and feedback collated. The decision to approve access will be the responsibility of the Registry Data Release Panel, which need to be unanimously in favour of the application to allow data to be released.

Before identifiable data are released to third parties, a signed Data Sharing Agreement is required following confirmation of the relevant permissions in all situations other than release of data to consultants or GPs relating to their own treated individuals (and upon receipt of a signed letter of request). In some cases, release of identifiable data will require completion of an application form and proof of approval from appropriate Research Ethics Committees and confirmation of Section 251 support from the NHS Health Research Authority or proof of informed consent.

A copy of the Data Access Request Form and more information can be found on the website (https://ysrccyp.org.uk/research/data-requests/).

## Ethics approval

The research work of the Register is undertaken with full ethical approval. Approval was originally obtained from the Northern and Yorkshire MREC (Ref. MREC/0/1/3) in May 2000 and amendments submitted for approval thereafter. The Yorkshire Register also has Health Research Authority Confidentiality Advisory Group approval, which provides a legal basis for processing identifiable data for the purpose of research via Section 251 of the NHS Act 2006 (Ref. 20CAG0133).

## Supplementary Material

dyac195_Supplementary_Data

## Data Availability

See ‘Data resource access’, above. The data are not publicly available due to privacy or ethical restrictions. The data that support the findings of this study are available on request from the corresponding author (subject to review, with the appropriate ethical and information governance approvals).
