# Supplementary material for: Data Resource Profile: Yorkshire Specialist Register of Cancer in Children and Young People (Yorkshire Register)
Source: Int J Epidemiol. 2022 Oct 13;52(1):e18–26. doi: 10.1093/ije/dyac195 (PMC9908036; doi:10.1093/ije/dyac195)
Supplement: dyac195_Supplementary_Data [file dyac195_supplementary_data.docx]

**Data Resource Profile: Yorkshire Specialist Register of Cancer in Children and Young People (Yorkshire Register) - Supplementary material**

**Supplementary Box S1**

Townsend deprivation scores were derived by applying National Census data^1^ based on residential postcode at the time of diagnosis to the cohort. We assigned deprivation score to each individual according to the lower super output area (LSOA) of residence at the time of diagnosis, using the latest index that was available preceding their time of diagnosis. Townsend score is stratified into population-weighted fifths (I-V)^2^ based on the total population of England at the time of each pertaining census^1^ for descriptive purposes in Table 1.

**Supplementary Box S2**

Yorkshire and Humber NHS Trusts*

•Airedale NHS Foundation Trust

•Bradford Teaching Hospitals NHS Foundation Trust

•Calderdale and Huddersfield NHS Foundation Trust

•Harrogate and District NHS Foundation Trust

•Hull and East Yorkshire Hospitals NHS Trust

•Mid Yorkshire Hospitals NHS Trust

•North Lincolnshire and Goole NHS Foundation Trust

•Sheffield Teaching Hospitals NHS Foundation Trust

•South Tees Hospitals NHS Foundation Trust

•York Teaching Hospital NHS Foundation Trust

**References**

1. Norman, Paul; “Area characteristics: Great Britain 1971 to 2011”, Mendeley Data, V1. 2017; doi: 10.17632/389scnndjy.1
2. Norman P. Identifying Change Over Time in Small Area Socio-Economic Deprivation. Applied Spatial Analysis and Policy. 2009;3(2-3):107-138.
